# Spinocerebellar Ataxia in a Hungarian Female Patient with a Novel Variant of Unknown Significance in the *CCDC88C* Gene

**DOI:** 10.3390/ijms24032617

**Published:** 2023-01-30

**Authors:** Fanni Annamária Boros, László Szpisjak, Renáta Bozó, Evelyn Kelemen, Dénes Zádori, András Salamon, Judit Danis, Tibor Kalmár, Zoltán Maróti, Mária Judit Molnár, Péter Klivényi, Márta Széll, Éva Ádám

**Affiliations:** 1 Department of Neurology, University of Szeged, 6720 Szeged, Hungary; 2 Department of Dermatology and Allergology, University of Szeged, 6720 Szeged, Hungary; 3 ELKH-SZTE Dermatological Research Group, Eötvös Loránd Research Network, 6720 Szeged, Hungary; 4 HCEMM-USZ Skin Research Group, University of Szeged, 6720 Szeged, Hungary; 5 Department of Immunology, University of Szeged, 6720 Szeged, Hungary; 6 Genetic Diagnostic Laboratory, Department of Pediatrics and Pediatric Healthy Care Center, University of Szeged, 6720 Szeged, Hungary; 7 Institute of Genomic Medicine and Rare Disorders, Semmelweis University, 1082 Budapest, Hungary; 8 Department of Medical Genetics, University of Szeged, 6720 Szeged, Hungary; 9 ELKH-SZTE Functional Clinical Genetics Research Group, Eötvös Loránd Research Network, 6720 Szeged, Hungary

**Keywords:** SCA40, CCDC88C mutation, JNK pathway, apoptosis, ataxia

## Abstract

Spinocerebellar ataxia (SCA) 40 is an extremely rare subtype of the phenotypically and genetically diverse autosomal dominant ataxias caused by mutations of the *CCDC88C* gene. Most reported cases of SCA40 are characterized by late-onset cerebellar ataxia and variable extrapyramidal features; however, there is a report of a patient with early-onset spastic paraparesis as well. Here, we describe a novel missense *CCDC88C* mutation (p.R203W) in the hook domain of the DAPLE protein encoded by the *CCDC88C* gene that was identified in a female patient who developed late-onset ataxia, dysmetria and intention tremor. To explore the molecular consequences of the newly identified and previously described *CCDC88C* mutations, we carried out in vitro functional tests. The *CCDC88C* alleles were expressed in HEK293 cells, and the impact of the mutant DAPLE protein variants on JNK pathway activation and apoptosis was assessed. Our results revealed only a small-scale activation of the JNK pathway by mutant DAPLE proteins; however, increased JNK1 phosphorylation could not be detected. Additionally, none of the examined mutations triggered proapoptotic effect. In conclusion, we identified a novel mutation of the *CCDC88C* gene from a patient with spinocerebellar ataxia. Our results are not in accord with previous observations and do not support the primary role of the *CCDC88C* mutations in induction of JNK pathway activation in ataxia. Therefore, we propose that *CCDC88C* mutations may exert their effects through different and possibly in much broader, yet unexplored, biological processes.

## 1. Introduction

Autosomal dominant cerebellar ataxias, also known as spinocerebellar ataxias (SCAs) are a group of progressive hereditary neurodegenerative disorders with significant clinical and genetic diversity [1,2]. Currently more than 40 subtypes of SCA have been distinguished. Spinocerebellar ataxia type 40 (SCA40) was first described in a Chinese family suffering from adult-onset cerebellar ataxia [3]. A missense allele of the coiled-coil domain-containing protein 88C (*CCDC88C*) gene was identified as a putative causative gene. Located in the long arm of chromosome 14, this gene encodes the Dvl-associating protein, which exhibits a high frequency of leucine residues (DAPLE) [3]. In addition to SCA40, mutations of this gene were proposed to be causative for nonsyndromic congenital hydrocephalus type 1 in an autosomal recessive manner [4].

Recently, the phenotypic spectrum of SCA40 has been expanded with new cases. The second reported incidence was identified in a Polish family in which members exhibited the clinical symptoms of hand tremor at rest and in action, slight ataxia, parkinsonism and cognitive decline [5]. Yahia et al. published a case study on a Sudanese patient with childhood-onset spastic paraparesis without cerebellar signs caused by another missense mutation of the *CCDC88C* gene [6]. An additional seven SCA40 patients of Kurdish, Chinese and Indian ancestry with dominantly cerebellar signs and variable movement disorders have been reported [7,8,9,10]. Segregation analysis clearly proved the putative disease-causing effect of the identified variants in four cases (p.R464H, p.D43N, p.E665K and p.R197Q). This analysis was not performed in two families (p.S1974R and p.F1024L), and analysis results were controversial in one case (p.R629Q) [3,5,6,7,8,9,10].

The effect of three identified missense mutations (p.R464H, p.D43N and p.E665K) was confirmed with in vitro functional analyses demonstrating that mutant (MT) DAPLE triggers c-Jun N-terminal kinase 1 (JNK1) activation and caspase-3 apoptotic signaling in cells [3,5,6]. However, the pathological effect of further four identified *CCDC88C* gene mutations (p.S1974R, p.R197Q, p.R629Q and p.F1024L) were not verified with functional analyses.

In this work, we describe the clinical features of a Hungarian spinocerebellar ataxia patient carrying a heterozygous novel *CCDC88C* missense mutation and compare it with the phenotypes of the previously reported SCA40 subjects. Furthermore, we report results of in vitro functional tests aimed at confirming the causative role of this new *CCDC88C* variant.

## 2. Results

### 2.1. Case Presentation

**Figure 1:**
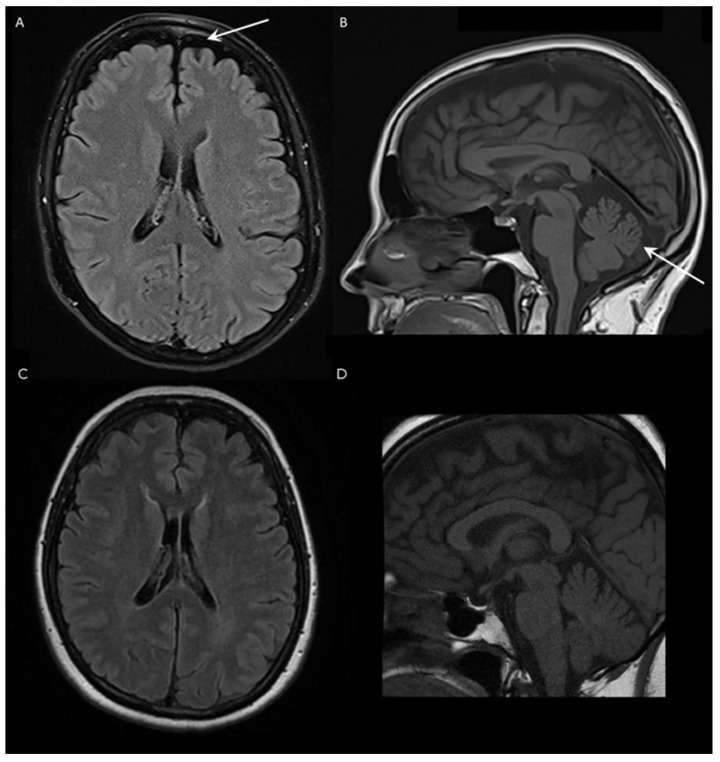
Brain magnetic resonance imaging (MRI) of a Hungarian SCA40 patient and an age-matched healthy subject. (**A**) Fluid-attenuated inversion recovery (FLAIR) axial and (**B**) T1-weighted sagittal scans of the patient. The arrows indicate the frontal lobe and cerebellar atrophy, respectively. (**C**) FLAIR axial and (**D**) T1-weighted sagittal images of a healthy age-matched female.

The first complaints of the patient presented in this report were episodic gait disturbance and dizziness at the age of 62. These problems first occurred only in the mornings, the duration of the episodes were 1.5 h; within 2 years, these episodes became longer and sometimes occurred during the afternoon as well. She had divergent strabism from childhood, as well as lumbar discopathy and hypothyroidism, due to Hashimoto thyroiditis. No similar gait difficulty was reported in her family. The neurological examination revealed strabism divergent on the left, horizontal gaze-evoked nystagmus, mild bilateral hypoacusis, slightly excavated feet, moderate spasticity in the ankles, mild paresis in both peroneal muscles, slightly slower fast alternating movements in the feet, mild gait ataxia, dysmetria and intention tremor in the upper limbs, slight ataxia in the lower extremities and discrete vibration hypesthesia in the feet. Dysarthria, ocular dysmetria, hyperreflexia, Babinski sign and extrapyramidal symptoms were not observed. The Scale for the Assessment and Rating of Ataxia (SARA) of the patient was 5 out of 40 in 2021, and the score did not progress during the following year. Nerve conduction studies did not detect polyneuropathy. Brain MRI examination showed mild cerebellar atrophy with vermian predominance and shrinkage of the frontal lobe with similar severity (Figure 1). Additionally, a small T2 hyperintense, T1 hypointense lesion was detected in the corpus callosum; however, its size decreased in the last 2 years. The spinal cord MRI did not demonstrate myelopathy. The video head impulse test revealed decreased vestibulo-ocular reflex on the left side and positional nystagmus on the right side. The cervical vestibular evoked myogenic potential test was absent on the right side and normal on the left side. Brainstem auditory evoked potential was normal. Detailed laboratory investigation did not detect obvious secondary etiology in the background of the ataxia. Serum levels of creatine kinase, albumin, B12, folic acid and alpha fetoprotein were all normal as well. The ANA, anti-TPO and antichromatin antibodies were positive in the last 2 years.

The patient was screened and found negative for Friedreich’s ataxia, SCA 1, 2, 3, 6, 7 and CANVAS by targeted genetic tests. Subsequently, clinical exome sequencing was performed, and a heterozygous missense variant was identified in the *CCDC88C* gene NM_001080414.3:c.607C > T, NP_001073883.2:p.R203W. The minor allele frequency of this mutation is 0.000029 according to the gnomAD database (https://gnomad.broadinstitute.org, Access date: 7 June 2022), and the mutation was predicted to be deleterious by SIFT, probably damaging by PolyPhen2 and disease-causing by Mutation Taster software. Segregation analysis was not performed because the parents of the proband were deceased. However, the patient reported that her parents did not have gait disturbance. Based on this observation, we hypothesized that the identified variant was possibly de novo; however, it is classified as variant of uncertain significance according to the ACMG guidelines. The patient also has two healthy children without neurological complaints who live abroad.

### 2.2. Expression of WT and MT Forms of CCDC88C Results in AP-1 Activation in HEK293 Cells

The role of the JNK pathway has been reported in different SCA conditions, and the presence of different variants of the *CCDC88C* gene has been associated with the disease. According to the reports, the activation of the pathway induces the hyperphosphorylation of JNK1 and triggers apoptosis [3,5,6,11].

To understand the effect of the newly identified genetic variant on the role of the encoded DAPLE protein in the disease, previously described in vitro experiments were carried out [3,5,6]. To monitor the effect of the novel mutation on the JNK pathway, HEK293 cells were transfected with the pcDNA3.1+/C-(K)DYK vector (GenScript) carrying the WT or MT *CCDC88C* R203W cDNAs. In this experiment, previously described mutations were also included as controls. The viability of the HEK293 cells was not affected by the transfection with any combinations of the plasmids, as it was assessed by MTT assay (Supplementary Figure S1), followed by regular microscopic control.

**Figure 2:**
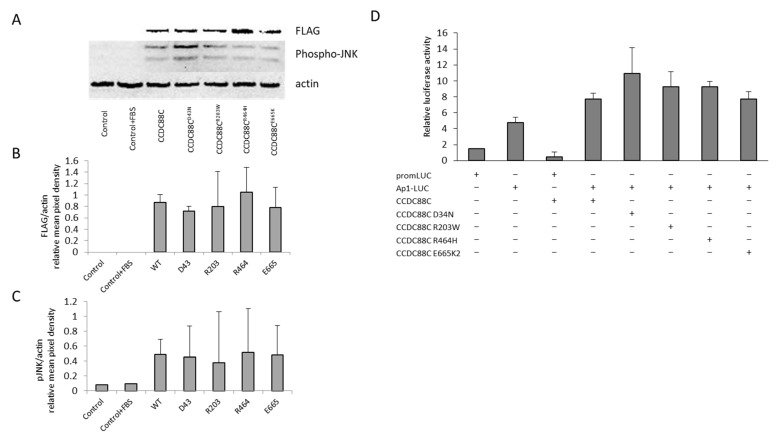
Overexpression of *CCDC88C* mutations induces slightly higher AP1–LUC activation. HEK293 cells were cotransfected with a plasmid construct containing FLAG-tagged wild type (WT) *CCDC88C* or one of the following mutants, (MT) *CCDC88C*: p.D43N, p.R203W, p.R464H and p.E665K, as well as by an AP-1 responsive element containing firefly-luciferase reporter construct. A Renilla luciferase construct was also included in each transfection reaction as an internal control, and promLUC and AP1–LUC vectors were also used as reporter controls. (**A**) Expression levels of WT and MT DAPLE-FLAG fusion proteins and endogenous protein levels of Phospho-JNK1 (P-JNK1) and actin were detected by Western blots and analyzed by densitometry (**B**,**C**). Anti-FLAG antibody was used for the visualization of DAPLE, antiphospho-JNK1 was used for visualization of endogenous P-JNK1 and antihuman-actin antibodies were used to demonstrate equal protein loading. Data are representative for five independent experiments. Control cells were maintained in Dulbecco’s Modified Eagle Medium (DMEM) with or without 10% fetal bovine serum (FBS). (**D**) Firefly luciferase activity was normalized to Renilla luciferase activity, and normalized luciferase activities were compared to the normalized luciferase activity of the promLUC transfected control samples. Data are represented as mean ± standard deviation (SD), *n* ≥ 3.

The expression levels of DAPLE proteins were estimated by Western blot analyses via detection of the FLAG epitope attached to the C-terminal end of the transfected transgenes. A comparable amount of the DAPLE proteins were detected in the transfected cells (Figure 2A,B).

We also analyzed JNK1 protein levels in control and transfected cells. We found no significant differences in the level of endogenous JNK1 between MT and WT *CCDC88C* transfected cells. Transfection of HEK293 cells with all constructs caused JNK1 phosphorylation. However, overexpression of the MT DAPLE proteins did not cause a change in the level of JNK1 phosphorylation in the cells, as compared to the effect of WT DAPLE (Figure 2A,C, Supplementary Figure S2).

Since these results contradict previously published data [3,5,6], we applied another widely used method that allows a more precise quantitation of the JNK pathway activation.

Phosphorylated JNK translocates to the nucleus, where it phosphorylates c-JUN, leading to the activation of the activator protein 1 (AP-1) transcription factor. Upon activation, AP-1 binds to the TPA responsive element (TRE) and induces transcription of a variety of genes involved in multiple cellular functions, such as proliferation, survival and differentiation. With an AP1–luciferase reporter construct, changes occurring in the AP1 pathway can be accurately monitored.

To evaluate how WT or MT forms of DAPLE affect transcription through AP-1 response elements, HEK293 cells were cotransfected with plasmids harboring WT or MT *CCDC88C* cDNAs and AP-1 firefly-luciferase reporter constructs, together with a plasmid expressing Renilla luciferase.

Luciferase activities were measured 24 h after transfection and normalized to transfection efficiency according to internal Renilla luciferase control. To determine basal luciferase activity, cells transfected with a vector carrying only a minipromoter—or AP-1 element—luciferase (promLUC or AP1–LUC) were used as controls in each experiment.

Overexpression of all tested DAPLE protein variants transcribed from WT and MT *CCDC88C* transgenes increased AP-1 directed luciferase activity, indicating that a higher level of either of these proteins results in elevated transcription from a promoter containing the AP-1 element (Figure 2D). Overexpression of the MT DAPLE proteins repeatedly resulted in a slight but insignificant increase in AP-1 activation as compared to the effect of WT DAPLE. In this respect, the p.D43N mutation showed the most prominent tendency in the AP1–LUC induction (Figure 2D).

### 2.3. Expression of MT Forms of DAPLE Protein Do Not Cause an Increase in Apoptosis in HEK293 Cells

JNKs have been shown to be involved in the mediation of apoptotic signaling. Proteolytic cleavage of caspase-3 is a commonly used marker for apoptotic cell death. It has been reported that overexpression of DAPLE D43N, R464H and E665K MT proteins cause a significant increase in caspase-3 cleavage compared to the WT protein [3,5,6,12].

**Figure 3:**
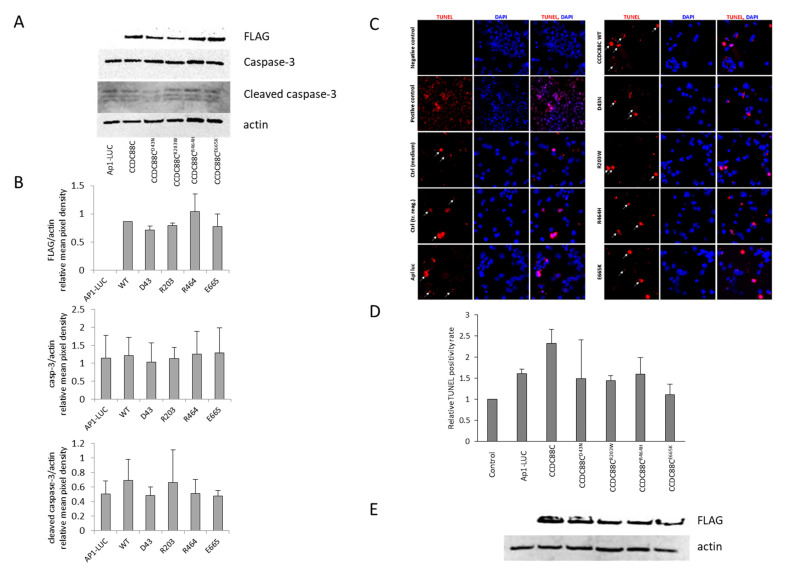
Overexpression of either WT or MT DAPLE forms does not trigger apoptosis in HEK293 cells. HEK293 cells were transfected with plasmid constructs driving expression of FLAG-tagged WT or p.D43N, p.R203W, p.R464H, and p.E665K MT DAPLE. (**A**) Expression levels of WT and MT DAPLE-FLAG fusion proteins, endogenous caspase-3, activated caspase-3 and actin were detected by Western blots (**B**) and analyzed by densitometry. Anti-FLAG was used for visualization of DAPLE, anticaspase-3 and anti-activated caspase-3 were used for visualization of endogenous caspase-3 and activated caspase-3 and antihuman-actin antibodies were used to demonstrate equal loading. (Caspase-3 could be detected only by loading 80 µg of total protein lysates.) The figure represents the results of five independent experiments. (**C**) In Situ Cell Death Detection Kit TMR red was used to detect the apoptotic cells. Representative images demonstrate TUNEL-positive nuclei (red color 40× magnification). Nuclei were visualized with 4’’,6-diamidino-2-phenylindole (DAPI) staining. Pictures were taken with a Zeiss Axio Imager Z1 microscope. (**D**) The rate of apoptotic cells was determined using the ImageJ software. Percentages (%) of TUNEL-positive cells relative to DAPI-positive total nuclei are indicated in the histogram. Data are presented as the mean of two independent experiments ± standard deviation; 700–1000 cells were counted for each construct per experiments. (**E**) Expression level of WT and MT DAPLE-FLAG fusion proteins was visualized by anti-FLAG and antihuman-actin antibodies to demonstrate equal protein loading.

To demonstrate whether expression of WT or MT forms of the *CCDC88C* gene results in an altered level of apoptosis, we investigated the activation of caspase-3 in transfected cells. Western blot analysis of total protein extracts isolated from transfected HEK293 cells showed similar DAPLE protein levels, and only very low amounts of cleaved caspase-3 were detected, regardless of whether the cells had been transfected or not (Figure 3A–C).

To gain more information about the apoptotic state of the cells, we chose to perform a TUNEL assay as it is a sensitive method for the detection of apoptotic cells. Overexpression of neither WT nor MT DAPLE protein caused an increase in the number of apoptotic cells (Figure 3C,D), whereas DAPLE protein expression levels were the same in cells overexpressing WT and MT DAPLE protein (Figure 3E).

## 3. Discussion

SCA40 is a hereditary neurodegenerative disorder with significant clinical and genetic diversity. The first report by Tsoi and colleagues described a *CCDC88C* missense mutation in an autosomal-dominant form of SCA40 [2]. Subsequently, similar results have been reported for additional missense mutations of the gene [5,6,7,8,9,10] (Supplementary Table S3). Interestingly, the CCDC88C mutation has also been found in 2010 to cause a complex hydrocephalic brain malformation in a large family [4]. Since this report, several new cases with these types of mutations have been identified [13,14], suggesting that these two conditions may be *CCDC88C*-related allelic disorders. Segregation analysis of four variants (p.R464H, p.D43N, p.E665K and p.R197Q) also indicate the pathogenic role of the *CCDC88C* mutations [3,5,6].

It was suggested that the described *CCDC88C* mutations may cause a loss of protein function through the truncation of binding motifs vital to the noncanonical Wnt pathway [4,15,16] or by modulating the phosphorylation status of the JNK pathway, thereby inducing caspase-3 cleavage and triggering apoptosis [3,5,6,17]. According to the literature, cellular functions, such as protein trafficking and cilium formation, are also affected by these mutations.

We identified a novel missense *CCDC88C* mutation (c.607C > T) resulting in an p.R203W substitution in the hook domain of the DAPLE protein in a Hungarian female patient presenting late-onset ataxia.

The clinical phenotype of the subject is characterized by slowly progressive episodic mild cerebellar symptoms, slight spasticity in the ankles and vibration hypesthesia in her feet. Similar neurological abnormalities were described in a Chinese family, although with more severe signs, which is supported by the difference in the SARA scores as well (Supplementary Table S1) [3]. Except mild spasticity in the ankles, no other upper motor neuron symptoms or movement disorders were noticed in the patient described in this case, although these neurological alterations were frequently observed in other cases [5,6,7,8,9,10]. Brain MRI examination showed mild cerebellar and frontal lobe atrophy, but no brainstem shrinkage, bilateral olivary degeneration or any other specific structural alterations were found.

To explore the molecular consequences of *CCDC88C* mutations, we expressed both the newly identified (p.R203W) and the previously described alleles (p.D43N, p.R464H and p.E665K) in HEK293 cells to examine JNK pathway activation.

WT and MT DAPLE proteins showed similar expression levels in the cells. Western blot analysis revealed no difference in JNK1 phosphorylation induced by WT or MT DAPLE proteins, and transfection of the cells by control plasmid constructs resulted in similar JNK1 phosphorylation. Furthermore, results from caspase-3 activation and detailed TUNEL assay did not support an increased proapoptotic effect of the novel and previously characterized *CCDC88C* mutations as compared to the WT allele, despite the fact that these experiments were performed by following the experimental design and conditions detailed in previous publications [3,5,6]. To further support these results, we included AP1–luciferase activity measurements. An increase in the luciferase reporter gene activation through an AP1 response element due to *CCDC88C* mutations—most prominently by p.D43N and, to a lesser extent, by p.R203W and p.R464H—was observed, indicating the activation of the JNK pathway.

In conclusion, we have identified a novel mutation of the *CCDC88C* gene in a patient with spinocerebellar ataxia. We found that DAPLE protein might be a positive regulator of the JNK pathway, and its mutations slightly but not significantly increased JNK induced AP1–LUC activity. At the same time, we observed that neither the novel *CCDC88C* allele nor the earlier described alleles of *CCDC88C* led to an increased rate of apoptosis through JNK1 hyperphosphorylation. Thus, our findings partly disagree with previous reports suggesting the role of DAPLE in ataxia by activation of the JNK pathway via dominant gain of function mutations.

Based on data from the literature and our own findings, we can conclude that (i) the pathogenic role of *CCDC88C* gene mutations in SCA40 is strongly supported by segregation analysis [2,4,5], (ii) the broad range of clinical symptoms in patients with SCA40 also depends on the site of the genetic mutation causing impaired function in different domains of the DAPLE protein (Supplementary Figure S3), (iii) *CCDC88C* mutations may exert their effect not only by affecting the JNK pathway but also by different and possibly in much broader biological processes and (iv) the development of the disease might be caused by mutations in *CCDC88C* and in other genes together, thus affecting not only JNK but different pathways as well. The interplay between a complex genetic background and the potential effects of harmful environmental factors could account for the diversity in both the age at disease onset and symptoms of the patients.

## 4. Materials and Methods

### 4.1. Clinical Examination

The patient was detected at the outpatient clinic of Department of Neurology University of Szeged by a movement disorder specialist having deep insight into ataxias and underwent a detailed diagnostic approach including neurological examination, laboratory and radiological investigations to exclude acquired causes of ataxia. After obtaining written informed consent, genomic DNA was extracted from peripheral blood leukocytes by standard protocol. First, the most common repeat expansion hereditary ataxias (SCA1, 2, 3, 6, 7, Friedreich’s ataxia and CANVAS) were tested. After the negative results of these tests, clinical exome sequencing was performed.

### 4.2. Cloning of the Constructs

The pcDNA3.1+/C−(K)DYK vector carrying the full length CCDC88C cDNA was purchased from GenScript Biotech Corp (Rijswijk, The Netherlands).

*CCDC88C* (NM_001080414.3, NP_001073883.2) (c.C607T), (c.G127A), (c.G1391A) and (c.G1993A) mutations were generated using the Quick Change Site-Directed Mutagenesis Kit (Agilent, Santa Clara, CA, USA), according to the instructions of the manufacturer. Oligoes used for the mutagenesis are listed in Supplementary Table S1. The mutations were introduced into the CCDC88C pcDNA3.1+/C−(K)DYK vector by replacing wild type (WT) sequences with mutated ones.

The miniprom−LUC and AP1–LUC cis-reporter plasmids were created by inserting the synthesized minipromoter and three copies of the AP1 response element (Supplementary Table S2) into the pGL4,201/luc2/Puro plasmid vector (Promega, Madison, WI, USA).

The final constructs carrying the MT CCDC88C cDNAs or the minipromoter and AP1 response element were verified by sequencing. Plasmid DNAs for transfection were purified with the QIAGEN Plasmid Maxi Kit (QIAGEN, Hilden, Germany).

### 4.3. Transfection of HEK293 Cell Line

The HEK293 cell line (Merck, Darmstadt, Germany) was maintained in Dulbecco’s Modified Eagle Medium (DMEM) supplemented with 10% fetal bovine serum (FBS, Lonza, Basel, Switzerland), 1% L-glutamine (Lonza) and 1% antimycotic–antibiotic solution (Lonza) at 37 °C in a humidified atmosphere with 5% CO_2_.

For transfection, cells were seeded into 12-well plates at a density of 300,000 cells/mL in full medium. After 48 h, medium was changed to FBS-free medium, and cells were co-transfected with the AP1–LUC cis-reporter plasmid, the pGL4.75 (hRluc/CMV) plasmid (Promega), which was used as internal control, and 1µg pcDNA3.1+/C-(K)DYK vector (GenScript) carrying the WT or mutant CCDC88C cDNA sequences. Transfection was carried out with the Lipofectamine 3000 transfection reagent (Thermo Fisher Scientific, Waltham, MA, USA), according to the manufacturer’s instructions. Mock-transfection by transfection reagent and cotransfection of luciferase plasmids with the empty pcDNA3.1(+) plasmid served as control. Twenty-four hours after transfection, cell samples were collected for extraction of proteins, which were subjected to luciferase activity measurement or to TUNEL assay.

### 4.4. Cell Viability Test

The viability of transfected HEK293 cells was monitored by regular microscopic control and MTT assays.

To assess whether cell viability was affected by transfection, cells were seeded into 96-well plates at a density of 150,000 cells/mL. After 48 h, medium was changed to FBS-free medium, and cells were cotransfected with the pcDNA3.1+/C−(K)DYK vector carrying WT or MT CCDC88C cDNA sequences or with the pAP1–LUC cis-reporter plasmid using the Lipofectamine 3000 transfection reagent (Thermo Fisher Scientific), according to the manufacturer’s instructions. Twenty-four hours after transfection, 3-(4,5-dimethylthiazol-2-yl)-2,5-diphenyltetrazolium bromide (MTT, Merck) was added to a final concentration of 0.5 mg/mL, and cells were incubated at 37 °C in a humidified atmosphere with 5% CO_2_ for 4 h. Subsequently, the medium was discarded, and the formazan crystals that had formed were solubilized in acidified isopropanol (20 mL 1N HCl and 500 mL isopropanol) supplemented with 2% SDS. Optical density (OD) was measured on a SPECTROstar Nano spectrophotometer (BMG Labtech, Ortenberg, Germany) at 540 nm. OD values were compared to the control wells transfected by the empty pcDNA3.1(+) vector and presented as a percentage (%) of living cells (Supplementary Figure S1).

### 4.5. AP-1 Luciferase Reporter Assay

To determine luciferase activity, cells were rinsed in phosphate-buffered saline (PBS) and lysed in passive lysis buffer (Promega).

Luciferase activity of the lysates was measured using the Firefly & Renilla Dual Luciferase Assay Kit (Promega) and a Synergy HTX multimode reader (Agilent, Santa Clara, CA), according to the manufacturer’s instructions. Luciferase activity derived from AP1–LUC plasmid was normalized to the activity of Renilla luciferase activity from the pGL4.75 (hRluc/CMV) plasmid.

### 4.6. Western Blot Analysis

Cells were washed twice with PBS, and protein was extracted by lysing the cells in lysis buffer containing 20 mM HEPES, 150 mM KCl, 1 mM MgCl2, 1 mM DTT, 0.5% Triton-X-100, 10% glycine, 0.1% NP-40 and 0.5% sodium dodecyl sulfate (SDS) (all chemicals were obtained from Merck) supplemented with 1% HALT™ Protease-Phosphatase Inhibitor 100X (Thermo Fisher Scientific). Samples were incubated for 30 min on ice with occasional vortexing, and cell debris was removed by centrifugation at 16,000 g for 10 min at 4 °C. The protein concentration of each sample was measured with the BCA-Kit (Thermo Fisher Scientific), and equal amounts of total protein were separated onto a 10% SDS polyacrylamide gel (SDS-PAGE) and blotted onto polyvinylidene difluoride membranes (Thermo Fisher Scientific).

After blocking with 5% milk in Tris-buffered saline with 0.05% Tween^®^ 20 detergent (TBST), membranes were probed with monoclonal mouse anti-FLAG antibody (1:1.000, Merck) for visualization of the (K)DYK-tag. Total and phospho-JNK proteins were detected using anti-JNK 3708 (1:1.000, Cell Signaling Technology, Danvers, MA, USA) and anti-p-JNK 5136 (1:500; Cell Signaling Technology) antibodies. Endogenous caspase-3 and cleaved caspase-3 were detected by anticaspase-3 and antiactivated caspase-3 antibody Asp175 (1:1.000 and 1:500; Cell Signaling Technology), respectively.

As a loading control, actin was visualized with the monoclonal antihuman actin antibody (1:1.000, Merck). Horseradish peroxidase-conjugated antimouse goat antibody and antirabbit goat antibody (Southern Biotech, Birmingham, AL, USA) were used as secondary antibodies. Chemiluminescent signals were detected and visualized on the Omega Lum G Chemidoc Imaging System (Aplegen Inc., Pleasanton, CA, USA). Before the membranes were used for probing with each antibody, previous antibodies were stripped by incubating the membranes in 0.1 M glycine at pH 1.9. Representative results of three individual experiments are shown.

### 4.7. TUNEL Assay

TUNEL method was applied to detect apoptosis of the HEK-293 cells. Cells were collected onto a microscopic slide using a cytocentrifuge (6 min, 600 RPM, 35,000 cells/slide) to create cytospin preparations. Cytospin samples were fixed in 4% paraformaldehyde for 20 min and then permeabilized on ice for 5 min in 0.1% Triton X-100 and 0.1% sodium citrate containing PBS. The In Situ Cell Death Detection Kit TMR red (Roche, Basel, Switzerland) was used to detect the apoptotic cells according to the manufacturer’s instructions. For the TUNEL reaction, one part enzyme and nine parts label solution were used for each sample, all of which were incubated for 60 min at 37 °C in a humidified chamber. One negative control (without the enzyme solution) and one positive control (digested with QIAGEN DNase I together with the TUNEL reaction) were applied for each experimental series. Nuclei were visualized with 4’’,6-diamidino-2-phenylindole (DAPI, Merck) staining. Five pictures were taken from randomly selected fields of each sample using a Zeiss AxioVert A1 microscope (20× original magnification, Carl Zeiss AG, Oberkochen, Germany). The rate (%) of TUNEL positive apoptotic cells was determined using the ImageJ software.

### 4.8. Statistical Analysis

Experiments were carried out in duplicate with at least three biological repeats. For statistical analysis one-tailed paired Student’s *t*-test was used with correction for multiple comparisons. The significance level was set at *p* ≤ 0.05.

## Data Availability

All data are presented in the manuscript.

## Supplementary Materials

The following supporting information can be downloaded at: https://www.mdpi.com/article/10.3390/ijms24032617/s1.
