# Supplementary material for: Spinocerebellar Ataxia in a Hungarian Female Patient with a Novel Variant of Unknown Significance in the CCDC88C Gene
Source: Int J Mol Sci. 2023 Jan 30;24(3):2617. doi: 10.3390/ijms24032617 (PMC9916980; doi:10.3390/ijms24032617)
Supplement: Supplementary file 1 [file ijms-24-02617-s001.zip › Supplementary Table 3.pdf]

Supplementary Table S3. Clinical characteristics of SCA40 patients published in the literature.

	Family 1		Family 2				Family 3	Family 4	Family 5				Family 6	Family 7	Family 8
Nationality	Chinese		Polish				Sudanese	Kurdish	Chinese				Chinese	Indian	Hungarian
Mutation (DNA)	c.1391G>A		c.127G>A				c.1993G>A	c.5922T>G	c.590G>A				c.1886G>A	c.3072C>A	c.607C>T
Mutation (protein)	p.R464H		p.D43N				p.E665K	p.S1974R	p.R197Q				p.R629Q	p.F1024L	p.R203W
Patient No.	II-4	II-5	III-1	IV-1	IV-2	IV-3	F83-581	IV-3	I-2	II-1	II-5	III-1	II-3	-	-
Sex	F	M	F	F	M	M	F	F	M	F	F	F	M	M	F
Age (years)	65	62	84	63	59	53	48	27	72*	62	51	39	61	50	65
Age at onset (years)	43	42	NA	45	49	33	childhood	13	48	42	40	35	59	50	62
First symptom(s)	Gait ataxia, dysarthria	Gait ataxia, dysarthria	UL tremor	UL tremor	UL tremor	UL tremor	Gait problem (tip-toeing)	Hand tremor	NA	Unsteady gait, blurred vision	Ocular dysmetria	Hand tremor	Hand tremor	Unsteady gait	Gait ataxia, dizziness
Gait ataxia	+	+	NA	+	+	+	-	+	+	+	+	+	+	+	+
UL ataxia	+	+	NA	NA	NA	NA	-	+	NA	NA	NA	NA	+	NA	+ (mild)
LL ataxia	+	+	NA	NA	NA	NA	-	+	NA	NA	NA	NA	+	NA	+ (mild)
Ocular dysmetria	+	+	NA	-	-	-	-	-	NA	+	+	+	-	+	-
Dysarthria	+	+	NA	-	-	-	-	+	+	+	+	+	+	+	-
Rest tremor	-	-	+	+	+	+	-	-	+	+	+	+	+	-	-
Action tremor	-	-	+	+	+	+	-	+	NA	-	-	-	-	-	-
Intention tremor	+	-	+	-	+	+	-	+	NA	+	-	-	+	-	+ (mild)
Spasticity	-	+	NA	-	-	-	+	-	NA	-	-	-	+	+	+ (mild)
Brisk tendon reflexes	+	-	NA	-	+	-	+	+	NA	+	+	-	+	+	-
Parkinsonism	-	-	NA	+	+	+	-	-	+	+	-	-	+	-	-
Dystonia	-	-	NA	-	-	-	-	-	NA	+	+	+	+	+	-
Cognitive impairment	-	-	+	+	+	+	-	-	-	+	+	-	+	-	-
Other	-	Impaired vertical gaze	Head tremor	-	-	-	Paraparesis, Babinski sign, foot deformities	Voice tremor	-	Paroxysmal limb jitter, Babinski sign	-	Paroxysmal limb jitter	Chorea/ dyskinesia, dysphagia, anxiety, depression	-	Divergent strabism, dizziness
SARA (0-40 points)	24	22	NA	5	10	3	NA	14	NA	17	10	6	18	NA	5
MMSE (0-30 points)	NA	NA	NA	NA	NA	NA	NA	29	NA	21	20	30	20	NA	NA
ACE (0-100 points)	NA	NA	NA	79	84	82	NA	NA	NA	NA	NA	NA	NA	NA	NA
Brain MRI	Mild pontine and cerebellar atrophy, moderate WM lesions	Mild pontine and cerebellar atrophy, mild WM lesions	NA	Normal	Normal	Normal	Periventricular leukomalacia with ischemic foci (WM, cerebellum, pons)	Normal	Age-related brain atrophy	Severe cerebellar, mild pontine atrophy	Normal	Moderate cerebellar, mild pontine atrophy	Mild cerebellar and pontine atrophy	Cerebellar and brainstem atrophy, bilateral olivary degeneration	Mild cerebellar (mainly vermian), mild frontal atrophy
Nerve conduction studies	NA	NA	NA	NA	NA	NA	Normal	NA	NA	Demyelination of motor nerves	LL neuropathy	NA	NA	Normal	Normal
Reference	2		4				5	6	7				8	9	current MS

+: present, -: not present, *: died

ACE: Addenbrooke's Cognitive Examination, F: female, LL: lower limb, M: male, MMSE: Mini-Mental State Examination, MRI: magnetic resonance imaging, NA: not available, SARA: Scale for the Assessment and Rating of Ataxia, UL: upper limb, WM: white matter
