# Supplementary material for: Spinocerebellar Ataxia in a Hungarian Female Patient with a Novel Variant of Unknown Significance in the CCDC88C Gene
Source: Int J Mol Sci. 2023 Jan 30;24(3):2617. doi: 10.3390/ijms24032617 (PMC9916980; doi:10.3390/ijms24032617)
Supplement: Supplementary file 1 [file ijms-24-02617-s001.zip › Supplementary Tables 1. 2.pdf]

Supplementary Tables

Supplementary Table S1. Oligos used for mutagenesis

CCDC88C^{D43N} *(Asp43Asn)*	
Fwd	5'-GACTATGTACATGGATTTAGTG**A**ACGGCATCTTTTTGAACC-3'
Rev	5'-GGTTCAAAAAGATGCCGT**T**CACTAAATCCATGTACATAGTC-3'
CCDC88C^{R203W} *(Arg203Trp)*	
Fwd	5'-GGCTCATCGACCAG**T**GGGACGAGTGCACCG-3'
Rev	5'-CGGTGCACTCGTCCC**A**CTGGTCGATGAGCC-3'
CCDC88C^{R464H} *(Arg464His)*	
Fwd	5'-CGAATGTGCGTCCAGCC**A**CATCCTGAAGCTGGAG-3'
Rev	5'-CTCCAGCTTCAGGATG**T**GGCTGGACGCACATTCG-3'
CCDC88C^{E665K} *(Glu665Lys)*	
Fwd	5'-GAAAGTCGAGGCCCTG**A**AGCATGAGAGCCAGGG-3'
Rev	5'-CCCTGGCTCTCATGCT**T**CAGGGCCTCGACTTTC-3'

Supplementary Table S2. Oligos used for cloning

Mini prom BglII Fw:	5'-gatctTAGAGGGTATATAATGGAAGCTCGACTTCCa -3'
Mini prom Hind3 Rev:	5'-agcttGGAAGTCGAGCTTCCATTATATACCCTCTAa-3'
AP1-response element SacI Fwd:	5'-cTGAGTCAGTGACTCAGTGAGTCAGTGACTCAGTGAGTCAGTGACTCAGc-3'
AP1-response element XhoI Rev:	5'-tcgagCTGAGTCACTGACTCACTGAGTCACTGACTCACTGAGTCACTGACTCAgagct-3'
