# Supplementary material for: Spinocerebellar Ataxia in a Hungarian Female Patient with a Novel Variant of Unknown Significance in the CCDC88C Gene
Source: Int J Mol Sci. 2023 Jan 30;24(3):2617. doi: 10.3390/ijms24032617 (PMC9916980; doi:10.3390/ijms24032617)
Supplement: Supplementary file 1 [file ijms-24-02617-s001.zip › supplementary_figures.pdf]

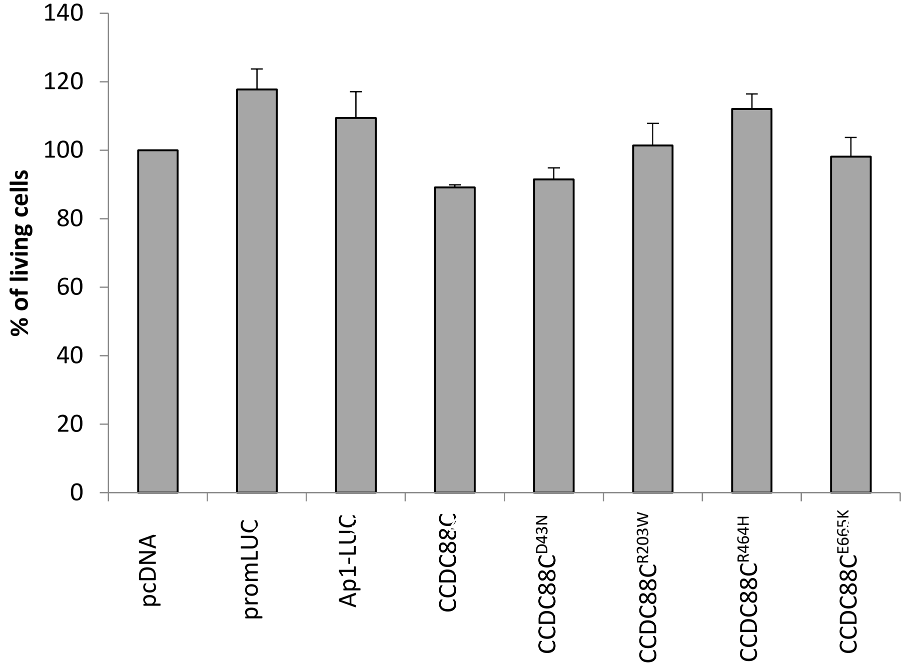

Supplementary Figure S1. Transfection with the chimerric constructs did not affect cell viability. MTT assay was performed in 96 well plates, OD values were compared to the empty vector transfected control cells (1st column), and results are presented as % of living cells. Statistical analysis was carried out by one-way ANOVA, not showing any statistical significance, n=3, data are presented as mean±SE.

A

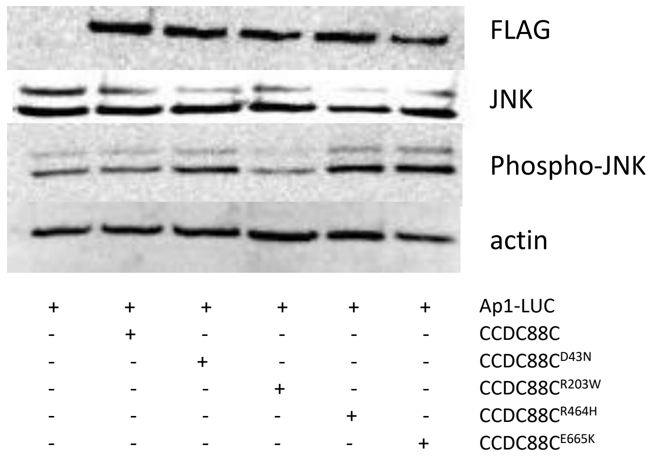

B

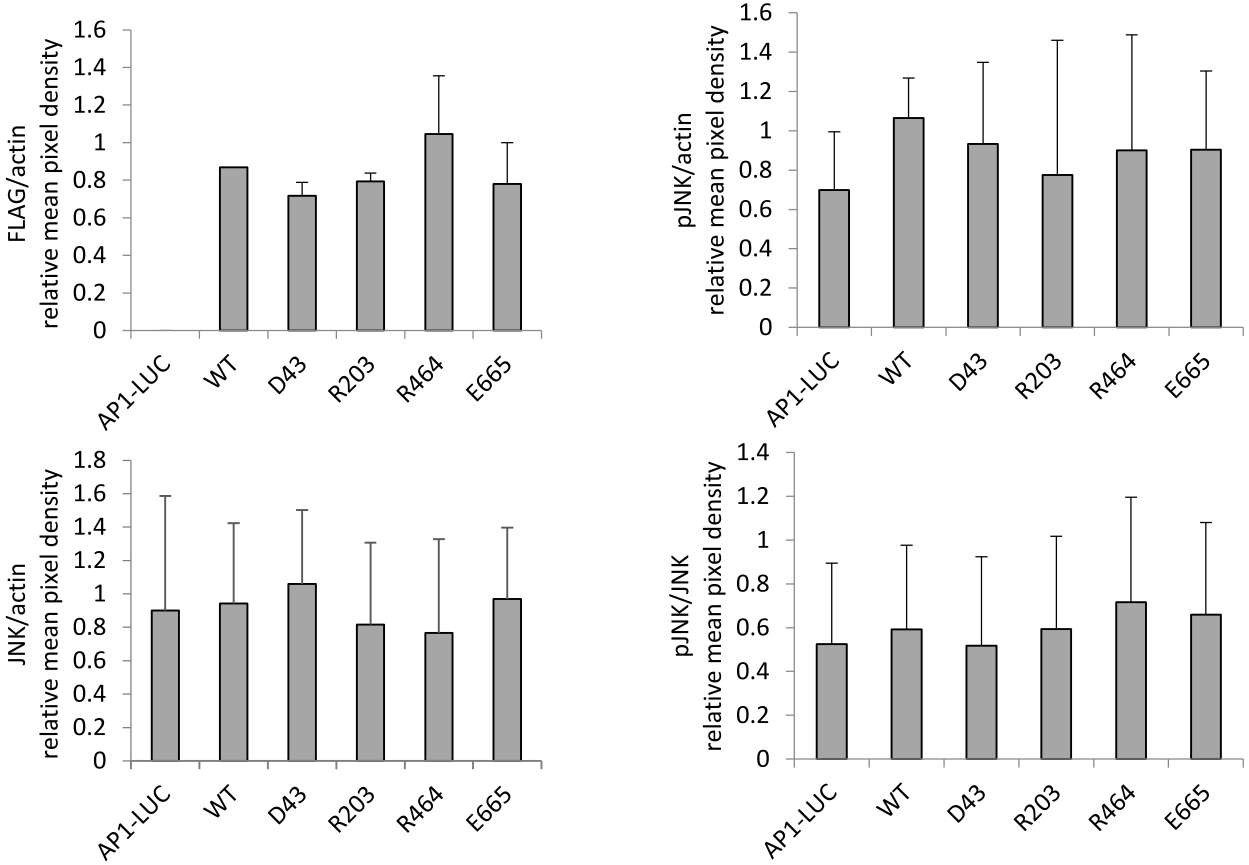

Supplementary Figure S2.

HEK293 cells were co-transfected with a plasmid construct containing FLAG-tagged wild type (WT) CCDC88C or one of the following mutants: (MT) *CCDC88C*: D43N, R203W, R464H,E665K, as well as an AP-1 responsive element containing firefly-luciferase reporter construct.

(A) Expression levels of WT and MT DAPLE-FLAG fusion proteins and endogenous protein levels of Phospho-JNK1 (P-JNK1) and actin were detected by western blots. Anti-FLAG antibody was used for the visualization of DAPLE, anti phospho-JNK1 was used for visualization of endogenous P-JNK1, and anti-human-actin antibodies were used to demonstrate equal protein loading. Data are representative for three independent experiments. (B) Densitometric analysis does not show significant differences in expression levels, based on data from 3 independent experiments.

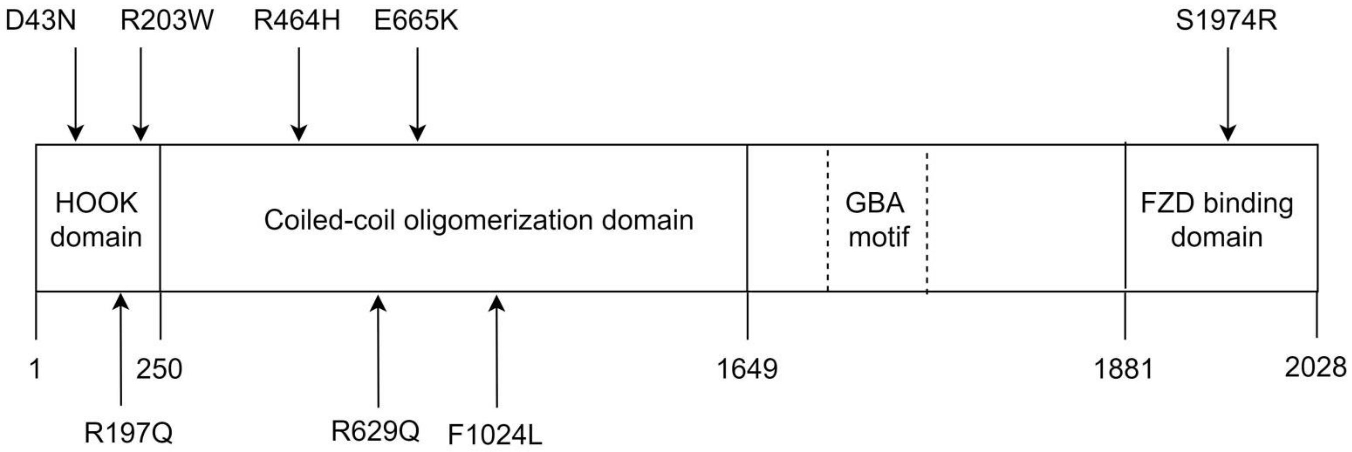

Supplementary Figure S3.
Schematic domain structure of the DAPLE protein
